# Complete Genome Sequence of the First Chinese Virulent Infectious Laryngotracheitis Virus

**DOI:** 10.1371/journal.pone.0070154

**Published:** 2013-07-29

**Authors:** Congcong Kong, Yan Zhao, Xianlan Cui, Xiaomin Zhang, Hongyu Cui, Mei Xue, Yunfeng Wang

**Affiliations:** 1 Division of Avian Infectious Diseases, State Key Laboratory of Veterinary Biotechnology, Harbin Veterinary Research Institute, The Chinese Academy of Agricultural Sciences, Harbin, China; 2 National Engineering Research Center of Veterinary Biologics, Harbin, China; 3 Animal Health Laboratory, Department of Primary Industries, Parks, Water and Environment, Prospect, Tasmania, Australia; 4 Institute of Animal Science and Technology, Yunnan Agricultural University, Kunming, China; Nanyang Technological University, Singapore

## Abstract

Infectious laryngotracheitis (ILT) is an acute respiratory disease caused by infectious laryngotracheitis virus (ILTV). The complete genome sequences of five attenuated ILTV vaccine strains and six virulent ILTV strains as well as two Australian ILTV field strains have been published in Australia and the USA so far. To provide the complete genome sequence information of ILTVs from different geographic regions, the whole genome of ILTV LJS09 isolated in China was sequenced. The genome of ILTV LJS09 was 153,201 bp in length, and contained 79 ORFs. Most of the ORFs had high sequence identity with homologous ORFs of reference strains. There was a large fragment deletion within the noncoding region of unique long region (U_L_) of ILTV LJS09 compared with SA2 and A20 strains. Though the origin binding protein of ILTV LJS09 existed, there was no AT-rich region in strain LJS09. Alignments of the amino acid sequences revealed seven mutations at amino acids 71 (Arg → Lys), 116 (Ala → Val), 207 (Thr → Ile) and 644 (Thr → Ile) on glycoprotein B, 155 (Phe → Ser) and 376 (Arg → His) on glycoprotein D and 8 (Gln→Pro) on glycoprotein L of ILTV LJS09 compared to those of virulent strain (USDA) as ILTV LJS09 did not grow on chicken embryo fibroblasts, suggesting the role of the key seven amino acids in determination of the cell tropism of ILTV LJS09. This is the first complete genome sequence of the virulent strain of ILTV in Asia using the conventional PCR method, which will help to facilitate the future molecular biological research of ILTVs.

## Introduction

Infectious laryngotracheitis virus (ILTV, *Gallid herpesvirus* 1) is an *alphaherpesvirus* that causes acute respiratory disease in chickens [1]. The clinical symptoms of infectious laryngotracheitis (ILT) depend on the virulence of a particular strain. Symptoms of ILT are characterized by nasal discharge, conjunctivitis, gasping, coughing, and expectoration of bloody mucus [2]. Although live attenuated ILTV vaccines have been used widely in China, ILT still occurs frequently. There is great concern within the poultry industry that current vaccines will fail to protect against newly evolved virulent field isolates or the vaccine strain will evolve to virulent strain [3], [4], [5], [6].

The complete genome sequences of five attenuated ILTV vaccine strains [7], [8], [9] and six virulent ILTV strains [10], [11] as well as two Australian ILTV field strains [12] have been published in Australia and the USA so far. A full genomic ILTV sequence was also assembled by concatenating partial sequences of six different ILTV strains [13]. Even though many Chinese virulent strains have been isolated and identified, the full genomic sequence of a Chinese virulent strain of ILTV has not been reported. To improve our understanding of ILTV virulence and mechanism associated with enhanced viral virulence, more information on the complete ILTV genome sequences and their genes is needed. In 2009, a virulent ILTV field strain, named LJS09, was isolated from diseased chickens in the southeast of China. In this study the first complete genome sequence of the Chinese strain LJS09 was determined using the conventional PCR method and sequencing.

## Materials and Methods

### Ethics Statement

All animal studies were approved by the Animal Ethics Committee of Harbin Veterinary Research Institute of the Chinese Academy of Agricultural Sciences (SYXK (Hei) 2011022). Care of laboratory animals and animal experimentation were conducted following “the Australian National Health and Medical Research Council Code of Practice for the Care and Use of Animals for Scientific Purposes” guidelines for housing and care of laboratory animals.

### Virus

ILTV LJS09 strain was isolated in 2009 from unvaccinated chickens in a farm in Jiangsu Province in China. The field sample was propagated in embryonated eggs as reported previously [5]. The trachea and its secretion from the infected chickens were homogenized with PBS (pH 7.4). After freeze-thaw three times, the mixture was clarified, filtered through a 0.22 µm filter, and treated with penicillin (500 U/ml) and streptomycin (500 U/ml). A suspension (200 µl) of the sample was inoculated into 9-day-old SPF embryonated chicken eggs via chorioallantoic membrane (CAM). Five days post inoculation, the CAM was harvested, homogenized and serially passaged five times [14].

### DNA Extraction and PCR Identification

Total DNA was extracted from homogenized CAM using the sodium dodecyl sulfate (SDS)-proteinase K and phenol/chloroform protocol [15]. A pair of primers within the glycoprotein B (gB) gene was designed to identify the genome. The nucleotide sequences of the forward and reverse primers of the gB gene of ILTV were 5′-TTCCGAGATCGAAGAAGTGAG-3′ and 5′-ACTCTGGTGGCAAGTATCCTGT-3′, respectively.

### Design of the Primers and Conventional PCR

A total of 102 pairs of primers (data not shown) were designed according to the ILTV Serva strain (accession number: HQ630064) to amplify the unique long (U_L_) and the unique short (Us) regions of ILTV LJS09 genome. Each pair of the neighbored primer was overlapped, and the amplified fragments were within 1500 bp. PCR was performed using 2 µl of template DNA (0.841 µg/µL) in total volume of 50 µl containing 5 µl of 10×Ex Taq buffer, 0.5 µl of Ex Taq (TaKaRa, Japan), 4 µl dNTPs (2.5 mmol/ml each), 2 µl of upstream primers (20 pmol/µl), 2 µl of downstream primers (20 pmol/µl), and ddH_2_O up to 50 µl. The thermal cycling conditions were as follows: 95°C for 5 minutes (1 cycle); 94°C for 30 seconds, 53–62°C for 30 seconds, 72°C for 90–150 seconds (30 cycles); 72°C extension for 10 minutes (1 cycle).

### Determination of the TR_S_/U_L_ Junction

The single primer PCR was used to determine the terminal repeat region (TR_S_) and the U_L_ junction that was modified according to the single oligonucleotide nested PCR (SON-PCR) [16]. The single primer was located in the 5′-terminal end of U_L_ region. Different from the SON-PCR, only one specific single primer and one pair of nested primers were used to amplify and identify the specificity of the products in this study, respectively. Compared with thermal asymmetric interlaced PCR (TAIL-PCR), the three reactions for SON-PCR contained only one specific primer and consisted of only two rounds of high-stringency amplification and one intermediate low-to-high temperature-ramping step. The first round of high-stringency PCR allowed the primer to drive the linear amplification of the DNA template of interest as single-stranded DNA. The low-to-high temperature-ramping step allowed the binding of that same primer to multiple partially complementary strands. Finally, the second round of high-stringency PCR led to the exponential amplification of the DNA of interest [16]. One specific primer was used in this study, which was located in the 5′ terminal end of the U_L_ region. The nucleotide sequence of the primer was 5′-GCGAGGTAGGGAGTG TGGCTGCTG-3′ (named SP-U_L_-5′). The protocol of modified single primer PCR is shown in Table 1.

**Table 1:** The protocol of modified single primer PCR.

Step	Temperature	Time	Cycles
1	95°C	5 min	1
2	94°C	30 s	10 cycles from step 2 to 4
3	60°C	30 s	
4	72°C	2 min	
5	94°C	30 s	1
6	28°C	2 min	1
7	Ramp to 72°C	2°C/s	1
8	72°C	2 min	1
9	94°C	20 s	30 cycles from step 9 to 11
10	59.5°C	30 s	
11	72°C	2 min	
12	72°C	10 min	1
13	4°C	∞	

The sequences of the nested primers within the U_L_ region used for identification of the specificity of the amplified products were as follows: Forward: 5′-GGTCGGACATGAAA CCACAAGG-3′; Reverse: 5′-TGGGTGCTTGCCTGCATATACC-3′.

### Determination of the Sequences and Loci of IR_S_/TR_S_

**Figure 1:**
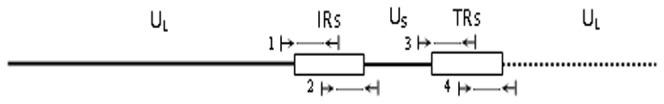
Schematic diagram of the locations of the primers designed to determine the sequences and loci of IR_S_/TR_S_. 1: The forward primer (U_L_-IR_S_ forward) located within the U_L_ region and the reverse primer (U_L_-IR_S_ reverse) located within the IR_S_ region. 2: The forward primer (IR_S_-U_S_ forward) located within the IR_S_ region and the reverse primer (IR_S_-U_S_ reverse) located within the U_S_ region. The U_L_-IR_S_ reverse and the IR_S_-U_S_ forward were overlapped. 3: The forward primer (U_S_-TR_S_ forward) located within the U_S_ region and the reverse primer (U_S_-TR_S_ reverse) located within the TR_S_ region. 4: The forward primer (TR_S_-U_L_ forward) located within the TR_S_ region and the reverse primer (TR_S_-U_L_ reverse) located within the U_L_ region. The U_S_-TR_S_ reverse primer and the TR_S_-U_L_ forward primer were overlapped.

Four pairs of primers were designed to determine the sequences and loci of internal repeat region (IR_S_) and TR_S_ (Figure 1). The nucleotide sequences of the primers were as follows (5′-3′): U_L_-IR_S_ forward: GTCAAATCTTTCTGCACGCGAC; U_L_-IR_S_ reverse: GTCAATCG GATCTTGTTCTGCAG; IR_S_-U_S_ forward: CCAGTTGAGAATCCCGACTCATC; IR_S_-U_S_ reverse: CTGTGTTTCCGACTCGGATGATG; U_S_-TR_S_ forward: GAAACCCACAAAC GAGCACGTC; U_S_-TR_S_ reverse: CAAAGAATC GTAGCGCCCACTC; TR_S_-U_L_ forward: TCATGTCCTCTGATTCCTCGAC; and TR_S_-U_L_ reverse: ACAGAAAGTAGGGGA GCGATTG.

### DNA Sequencing

PCR products were cloned into pMD18-T vectors (TaKaRa, Japan) and transformed into E. coli DH5α competent cells. Plasmid PCR was used to screen the positive clones. The positive clones were sequenced by Shanghai Invitrogen Biotechnology Co. Ltd (Shanghai, China). To guarantee the fidelity of results, at least three positive clones for each amplification product were sequenced for three times.

### Genome Assembly and Analysis

DNA sequences were assembled using the Seqman program (DNASTAR, Madison, WI) and mapped manually. The complete sequence of ILTV LJS09 was submitted to GenBank. Open reading frames were predicted by the NCBI ORF Finder program and GeneMark program [17]. Nucleotide and amino acid sequence alignments with the reference strains (Table 2) were performed using DNAMAN and Geneious software package.

**Table 2:** Comparison of full genome sequences of ILTV strains.

Strain	Pathotype	Accession No.	UL	IRS	US	TRS	Total length (bp)
1874C5	Virulent	JN542533	113030	12283	13094	12284	150691
USDA	Virulent	JN542534	109580	14547	13095	14547	151769
81658	Virulent	JN542535	109575	13833	13094	13833	150335
63140/C/08/BR	Virulent	JN542536	112915	13812	13094	13812	153633
Serva	Live attenuated	HQ630064	113930	12803	13094	12803	152630
SA2	Live attenuated	JN596962	114179	12835	13126	12835	152975
A20	Live attenuated	JN596963	114180	12836	13126	12836	152978
LT Blen	Vaccine	JQ083493	112801	13800	13093	13800	153494
Laryngo	Vaccine	JQ083494	112801	13800	13094	13800	153495
LJS09	Virulent	JX458822	112911	13598	13094	13598	153201

## Results

### PCR Identification of the Genome

**Figure 2:**
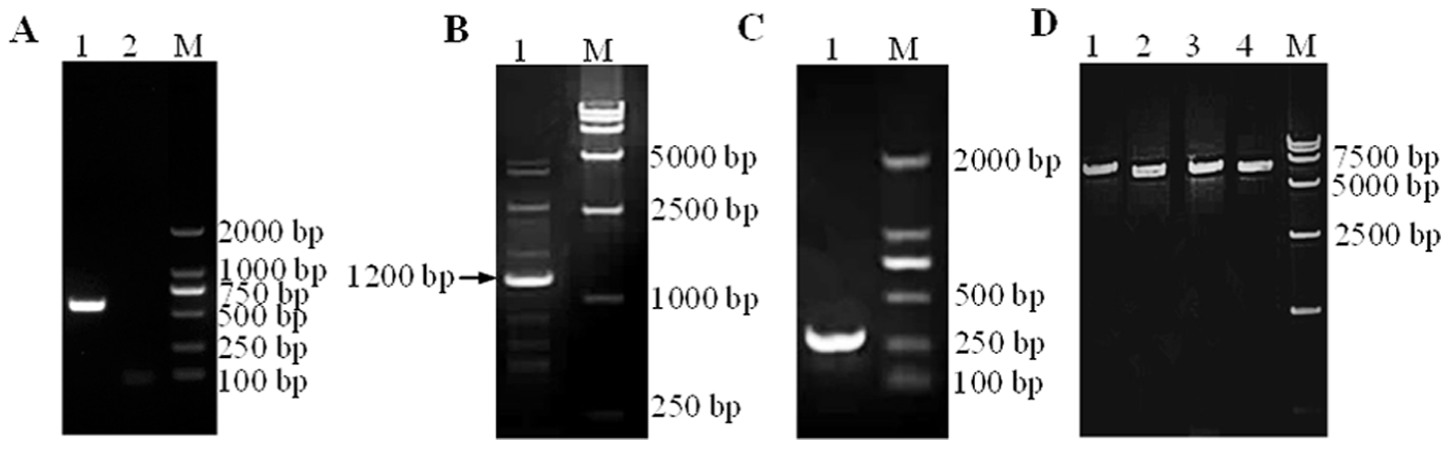
Ethidium bromide stained agarose gels of PCR amplified DNA. A: The PCR product amplified from the genomic DNA using the gB primers (Lane 1) and negative control (Lane 2). B: The single primer PCR products using the SP-U_L_-5′; C: The specificity of the product using the nested primers; D: The PCR products of IR_S_ and TR_S_. Lane 1: The PCR product amplified using the U_L_-IR_S_ forward and U_L_-IR_S_ reverse primers; Lane 2: The PCR product amplified using the IR-US forward and IR-US reverse primers; Lane 3: The PCR product amplified using the U_S_-TR forward and US-TR reverse primers; and Lane 4: The PCR product amplified using the TR-UL forward and TR-UL reverse primers.

The PCR product amplified using the gB primers with the genomic DNA templates was 567 bp long (Figure 2A) as expected and the sequence identity with other ILTV reference strains was up to 99% (data not shown).

### Determination of the TR_S_/U_L_ Junction

There were many PCR products (Figure 2B) from the single primer PCR and only one band in nested PCR (Figure 2C). Several faint products were detected (Figure 2B), and each of the products that were separated in the agarose gel was extracted and identified with the nested PCR primers. Only the product of a 1200 bp fragment was identified specific by the nested primers. Analysis of the nucleotide sequence of the product indicated that 255 bp of the 1200 bp was consistent with the U_L_ region and the remainder 945 bp was identical with the TR_S_ region of the reference sequence (strain Serva, Accession No. HQ630064). It was concluded that there was genome structures by which the TR_S_ region and the U_L_ region were connected.

### Determination of the Sequence and Locus of IR_S_/TR_S_

Four pairs of primers were designed to amplify the IR_S_ and TR_S_ regions. According to the results of the determination of the junction of the TR_S_/U_L_, there were genome structures through which the TR_S_ and U_L_ regions were connected. Based on this conclusion, the TR_S_ region was also determined by a pair of primers located in the TR_S_ and U_L_ regions respectively. The PCR products were 7211 bp, 6945 bp, 7037 bp and 7031 bp, respectively (Figure 2D).

### Genomic Organization

**Figure 3:**
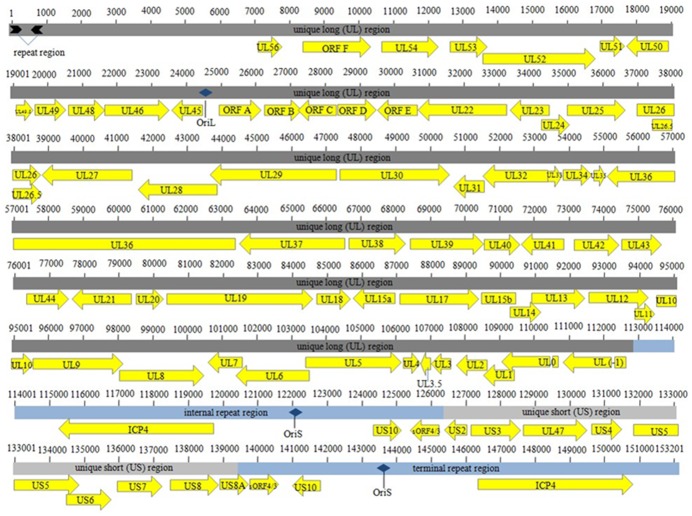
The concatenated genome map of ILTV strain LJS09. The locations and sizes of predicted ORFs, repeat regions and origins of DNA replication were annotated manually according to the annotated sequence of LJS09.

The genome of ILTV LJS09 was 153,201 bp in size, with a G+C content of 48.1%. The genome sequence of ILTV strain LJS09 has been submitted to the GenBank (accession no. JX458822). The LJS09 genome was organized with four genomic regions [18], [19]. The U_L_ region was 112,911 bp long. The unique short (U_s_) region (13,094 bp) was flanked by the TRs and IRs [18], [19] that was 13,598 bp each. The LJS09 genome contained 79 predicted ORFs that had high similarities with homologous ORFs of other ILTV-1 strains [7], [8], [9], [10]. An annotated genome map of the LJS09 strain is shown in Figure 3. Compared with the reference strains, there was a large fragment deletion in the non-coding region of the U_L_ region of LJS09 genome. The deletion was 189 bp long that was located between 3292–3480 bp of SA2 genome. The LJS09 genome had the highest similarity (99.6%) with that of the virulent strain 63140/C/08/BR while the identity with virulent strain USDA was the lowest (96.4%).

### Comparison of ORFs between LJS09 and Reference Strains

In order to identify the differences between LJS09 and reference strains, the amino acid sequences of these strains were aligned. Twenty genes (gC, UL3, UL4, UL11, UL12, UL13, UL14, UL20, UL24, UL25, UL26.5, UL31, UL32, UL33, UL34, UL35, UL40, UL45, UL49, UL51) showed 100% identity. All the reference strains had identical amino acids in the listed genes except LJS09 strain that had unique mutations (Table 3).

**Table 3:** Amino acid mutations unique to LJS09.

ORF	Amino acidposition	Conserved amino acid^a^*	LJS09	ORF	Amino acidposition	Conserved amino acid	LJS09
gB	71	R	K	UL18	120	N	D
gB	207	F	L	UL19	225	I	T
gD	155	F	S	UL19 784	784	Q	R
gD	376	R	H	UL19	785	V	A
gI	14	T	A	UL19	835	R	G
gI	109	E	V	UL19	888	L	S
gM	350	K	E	UL21	102	N	Y
ICP4	488	R	H	UL29	381	I	F
ICP4	491	R	C	UL30	480	L	P
ICP4	615	F	S	UL30	486	K	E
ICP4	1170	G	E	UL30	601	S	P
ICP4	1408	H	R	UL30	1040	L	F
ORF A	214	Y	H	UL36	1084	R	G
ORFC	161	I	T	UL36	1200	Y	F
ORFE	375	S	P	UL36	1531	A	V
sORF4/3	20	R	Q	UL36	1928	F	I
sORF4/3	131	N	S	UL36	2648	R	H
sORF4/3	255	K	M	UL37	473	P	A
UL5	223	I	T	UL37	886	A	V
UL5	384	K	R	UL18	120	N	D
UL7	85	E	G	UL19	225	I	T
UL9	214	E	G	UL19 784	784	Q	R
UL9	704	I	K	UL19	785	V	A
UL15	525	I	V	UL19	835	R	G

a *: the amino acids conserved in all of the reference strains showed in Table 2.

The amino acid residues AAQD at amino acids 87–90 in the infected cell polypeptide 4 (ICP4) gene are unique to TCO (tissue culture origin) vaccine strains.

### The OriS Region of Strain LJS09

**Figure 4:**
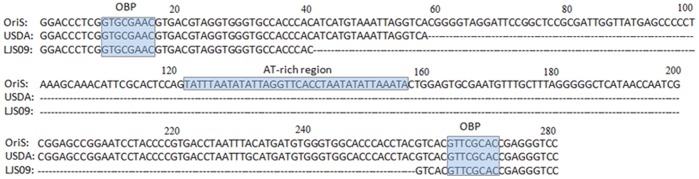
Nucleotide alignment of the origin of DNA replication (OriS) sequence (accession number: AM238250) with those of strains USDA and LJS09. OBP: origin binding protein. The OriS sequences of strains 1874C5, 81658, 63140/C/08/BR, Serva, SA2, A20, LT Blen, and Laryngo were identical to that of the OriS (accession number: AM238250).

Compared with the OriS sequence (accession no. AM238250), there was a deletion of 217 bp within the OriS region in LJS09 strain. There was a deletion of 76 bp in the OriS region of strain LJS09 when it was compared with the USDA strain (accession no. JN542534). Interestingly, both the origin binding protein (OBP) sites of USDA and LJS09 strains still existed. Neither the USDA strain nor the LJS09 strain contained the AT-rich region (Figure 4).

### Analysis of Mutated Amino Acids of ILTV Glycoproteins Associated with the Cell Tropism

The American virulent reference strain (USDA) could be propagated in CEF cells, but the Chinese strain LJS09 in this study could not. To locate the mutations associated with the cell tropism, amino acid sequences of glycoproteins gB, gC, gD, gH and gL of these two strains were analyzed. No mutations were found on gC protein. Seven amino acid mutations were found at 71 (Arg → Lys), 116 (Ala → Val), 207 (Thr → Ile) and 644 (Thr → Ile) on gB protein, at 155 (Phe → Ser) and 376 (Arg → His) on gD protein, and at 8 (Gln→Pro) on gL protein compared to those of strain USDA.

## Discussion

In this study, a pair of primers located in the gB gene was used to identify the ILTV. gB is a highly conserved gene of herpesvirus [20], but the homology between α-herpesviruses was low and it can be used as a target gene in the laboratory diagnosis of ILTV.

The genome of LJS09 was sequenced by the conventional method that was different from the high-throughput sequencing. In this study, both the sequences and loci of the repeat regions were determined precisely through conventional PCR and sequencing. To ensure the accuracy of the sequence, at least three clones were sequenced. The single oligonucleotide nested PCR can be used to identify any DNA region adjacent to a known one [16]. In this study, only one specific single primer was used to amplify the products, followed by purification and identification of each of the products by a pair of nested primers. The identified product consisted of the 5′ terminal sequence of U_L_ region adjacent to 3′ terminal sequence of TRs. The genomic DNA of ILTV is replicated through the rolling circle replication. In the process of DNA replication, the genomic DNA concatermer can be formed [21]. In this study, we confirmed the ILTV genome structure through which the TR_S_ and U_L_ region were connected. Therefore, it is feasible to determine the actual sequence of the 3′ terminal of TR_S_ and 5′ terminal of U_L_.

The genes gC, UL3, UL4, UL11, UL12, UL13, UL14, UL20, UL24, UL25, UL26.5, UL31, UL32, UL33, UL34, UL35, UL40, UL45, UL 49 and UL 51 may play no or only a minor role in ILTV virulence since there is no difference in the amino acid sequences of these genes between strains.

SA2 and A20 are TCO vaccine strains whereas Serva, Laryngo, LT Blen are CEO vaccine strains. Particularly, CEO vaccine strains are more easier to revert to virulence than TCO vaccine strains after bird-to-bird passage [22] or after reactivation from latency [23]. ICP4 is a regulator of viral transcription that is required for productive infection [24], [25] and the possibly flexible region of the protein allows it to efficiently interact with multiple transcription factors [26]. The expression level of ICP4 varies from the stress factors and the latency infection periods. Therefore, ICP4 is closely related to the reactivation of latent virus [27]. The insertion of the four amino acid residues in the ICP4 of TCO vaccine strains may affect the ability of ICP4 to active the transcription factors that may decrease the reactivation capacity of the latent virus. In addition, the difference of the four amino acid residues can be used to distinguish the TCO from the CEO besides the PCR-RFLP [3].

Glycoproteins play an important role in virus attachment to and penetration of host cells and are related to host range diversity [28]. Glycoproteins gB [29], [30], gC [31], [32], gD [33], [34] and gH/gL [35], [36], [37] are important for interaction between the virus and the host, and mutations of these genes may alter the structure and function of the corresponding protein, therefore affecting or inhibiting the interaction of the virus and the receptors on the host cells [38], [39]. Since the American virulent reference strain (USDA) could be propagated in CEFs but the Chinese strain LJS09 in this study could not, these unique mutations in ILTV LJS09 may indicate the importance of these amino acids for the cell tropism of ILTV LJS09. Amino acid sequence alignments revealed seven mutations at amino acids 71, 116, 207 and 644 on gB protein, 155 and 376 on gD protein and 8 on gL protein of ILTV LJS09 compared to those of strain USDA, suggesting the important role of these amino acids on gB, gD and gL proteins in determination of the cell tropism of ILTVs.

Like many other alphaherpesviruses, ILTV possesses three origins of viral DNA replication, with two copies of OriS located in the IR_S_ and TR_S_ regions and one copy of OriL located in the U_L_ region [40]. OriS contains a palindrome structure and studies on OriL (a palindrome of some 136 bp overall) of both HSV-1 and HSV-2 have shown that it is highly prone to deletion from plasmid clones carried in *Escherichia coli* [41]. In order to ensure the accuracy of the sequence, the PCR product of this region was sequenced in our study even though deletions of oriL or both copies of oriS have little effect on viral replication in vitro [42], [43]. The results of the OriS sequences may support these previous findings since a deletion was only found in OriS of virulent strains LJS09 and USDA but not in those of other virulent or attenuated vaccine strains.

Although several whole genomes of ILTV strains have been determined and reported, there are no reports of the whole genomes of the ILTV strains in Asia so far. This is the first complete genome sequence of ILTV isolated in China, which will facilitate the future study of molecular biology of ILTV.
